# A Case of Primary Endometriosis Associated With an Umbilical Hernia

**DOI:** 10.7759/cureus.27626

**Published:** 2022-08-03

**Authors:** Zanan A Odhar, Mustafa R Muhi, Hasanain A Odhar

**Affiliations:** 1 Department of Gynecology, Al Mahmoodyia General Hospital, Baghdad, IRQ; 2 Department of General Surgery, Al Mahmoodyia General Hospital, Baghdad, IRQ; 3 Department of Pharmacy, Al-Zahrawi University College, Karbala, IRQ

**Keywords:** umbilical reconstruction, umbilical hernia, primary umbilical endometriosis, umbilical endometriosis, endometriosis

## Abstract

Endometriosis is a common gynecological disease that mainly influences pelvic organs. However, extra-pelvic endometriosis is considered less prevalent. Here, we present a case study of umbilical endometriosis associated with an underlying hernia. It is known that the incidence of such a case is very rare and its diagnosis is challenging. Surgical excision of this type of lesion with a repair of the hernia is considered the preferred management approach. In this case, a 40-year-old female was admitted to the hospital with cyclical umbilical bleeding for one year. The bleeding would start with her menstruation and continue throughout the menstrual period. Initial diagnosis was made by physical examination and ultrasound imaging. Treatment options were discussed with her and she accepted to undergo surgical wide excision of the umbilical lesion with a repair of the hernia. Diagnosis was further confirmed by a histopathological study of the lesion tissue specimen. Also, a referral to a gynecologist did not report any abnormality or pelvic disease, and the patient was discharged well from the hospital.

## Introduction

Endometriosis is a chronic and mostly benign gynecological condition that influences up to 22% of females. It is characterized by the presence of endometrial glands as well as stroma outside the uterine cavity and musculature [1,2]. Generally, endometriosis influences pelvic organs leading to menorrhagia, dysmenorrhea, infertility and pelvic pain. Extragenital or extra-pelvic endometriosis is considered less common; however, it has been observed in various parts of the female body like bladder, bowel, brain, lungs, umbilicus, surgical scars [3]. Umbilical endometriosis is a very rare condition that accounts for only 0.5%-1% of all extra-pelvic endometriosis cases. It commonly develops secondary to surgical scars, but it may also present very rarely as spontaneous or primary umbilical endometriosis [4].

Several theories have been suggested to clarify the pathogenesis of endometriosis such as the embryonal rest theory, the coelomic metaplasia theory and the migratory pathogenesis theory. The first one, the embryonal rest theory, proposed that the remnants of the Wolffian duct or Müllerian duct are the precursors of ectopic uterine tissue while the coelomic metaplasia theory stated that certain inflammatory, hormonal or traumatic factors can stimulate the embryonic coelomic mesothelium to undergo transformation into endometrial tissue. But the most accepted theory is the migratory pathogenesis theory that assumed that the dissemination of endometrial tissue can take place by vascular and lymphatic channels, direct extension, and surgical manipulation [5]. During surgical operations, endometrial tissue can be implemented directly into surgical incisions. However, the exact pathogenesis of primary endometriosis is still less clear. For primary endometriosis, it is possible that the umbilicus can act as a physiologic scar with a tendency for implantation as endometrial cells course the lymph channels [6].

The usual frequency of umbilical hernias ranges from 3% to 8.5% of all cases of abdominal wall hernias [7]. However, the incidence of spontaneous umbilical endometriosis that is associated with an underlying hernia is considered very rare and it represents a diagnostic challenge in the medical field [2]. In this article, we report a case of primary endometriosis associated with an umbilical hernia.

## Case presentation

**Figure 1:**
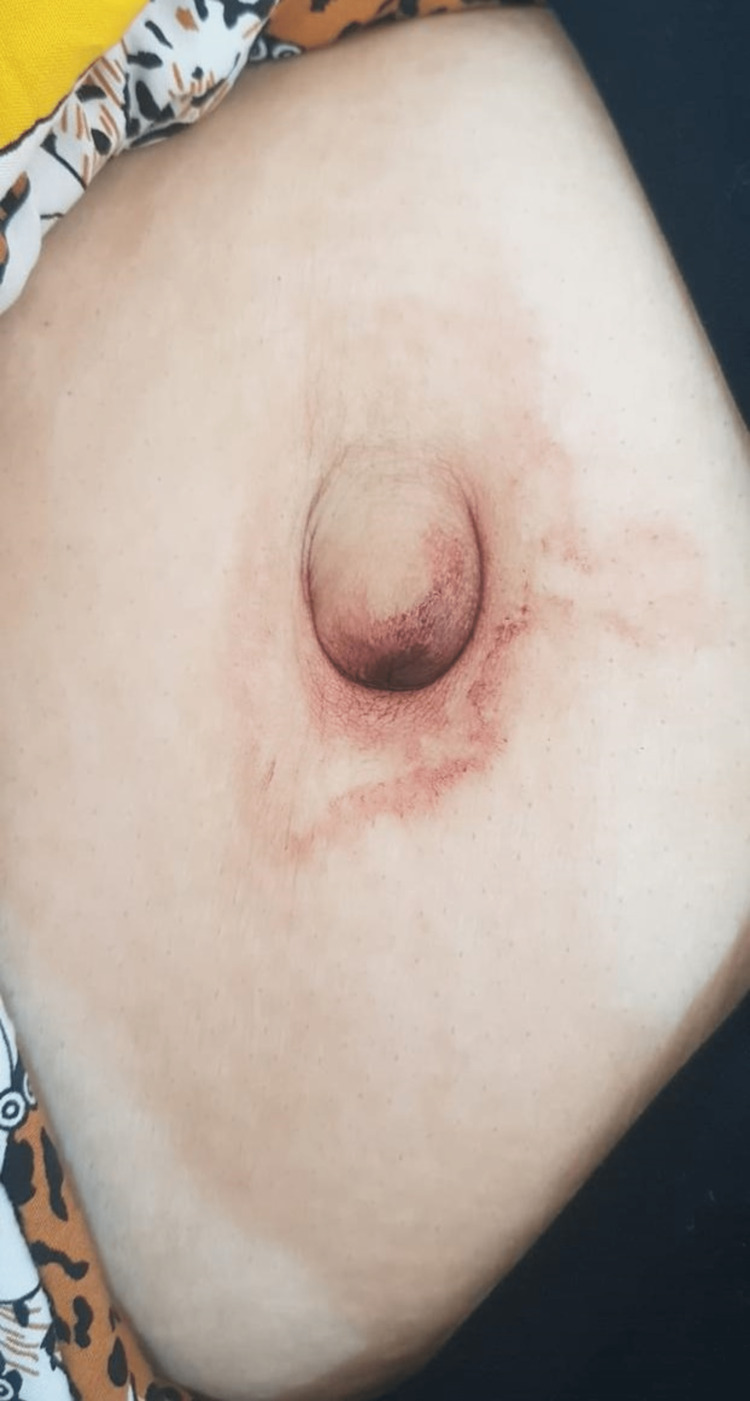
Umbilical bleeding coinciding with the patient’s menses

A 40-year-old female patient presented to the hospital with a symptom of cyclical umbilical bleeding for one year (Figure 1). This bleeding would begin with her menses and last throughout her period. The patient denied umbilical pain, dyspareunia, dysmenorrhea, infertility and any past history of endometriosis. The patient had previous two caesarean sections by a lower abdominal (Pfannenstiel) incision. Also, no abnormality was recognized in her blood work.

A physical examination was carried out to assess the umbilical nodule and associated hernia. Diagnosis was further confirmed by ultrasound imaging that referred to evidence of subcutaneous, sub-umbilical, hypo-echoic and heterogenous mass lesion (28 mm × 22 mm) with vascularity suggesting an endometrioma. Also, the ultrasound image report pointed to evidence of umbilical hernia with a neck of 14 mm; it was reducible and containing bowel. After discussing the possible management options, the patient agreed to undergo a primary hernia repair with excision of the subcutaneous mass and umbilical reconstruction.

**Figure 2:**
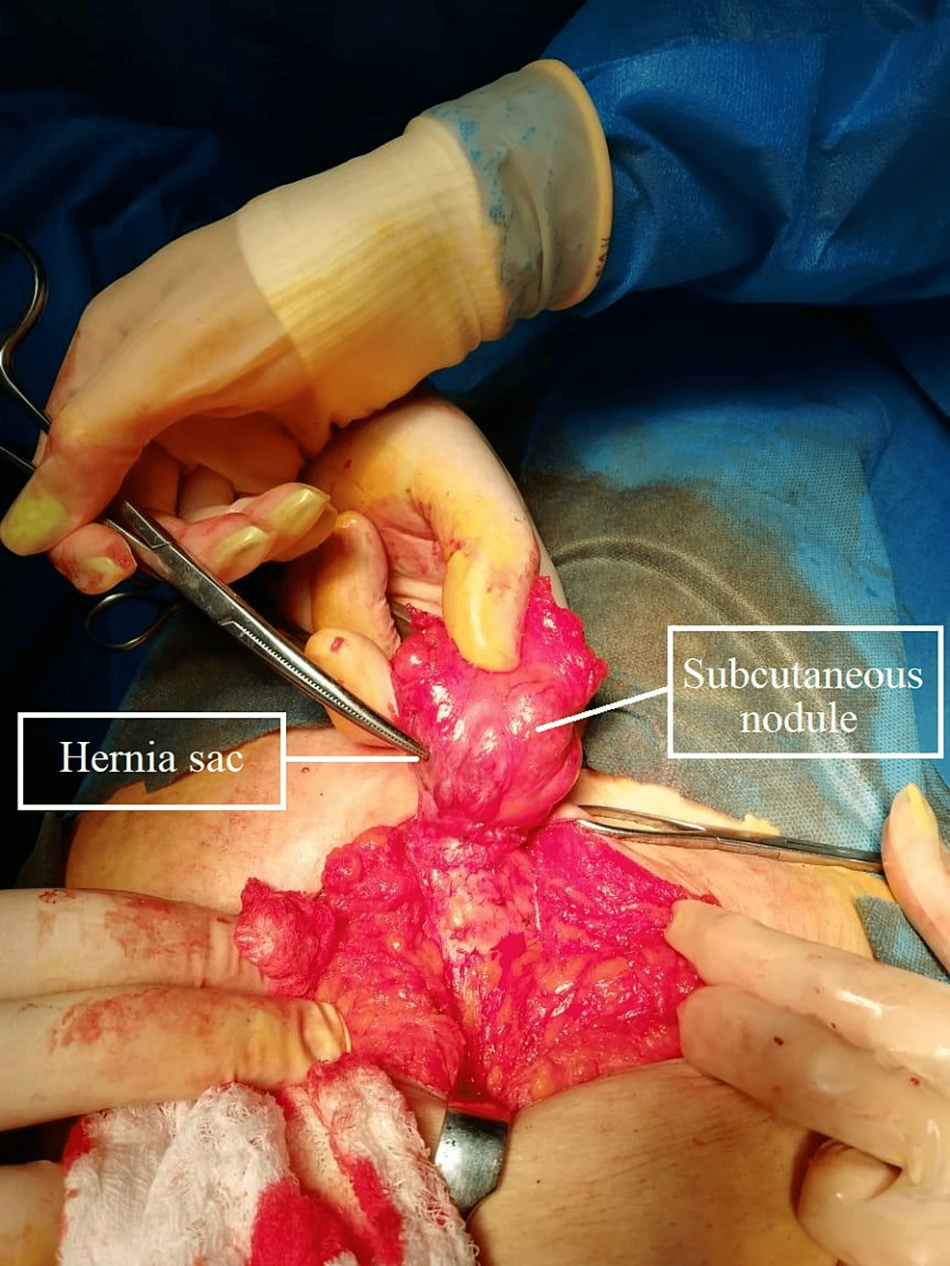
Intraoperative image of the subcutaneous nodule and hernial sac

**Figure 3:**
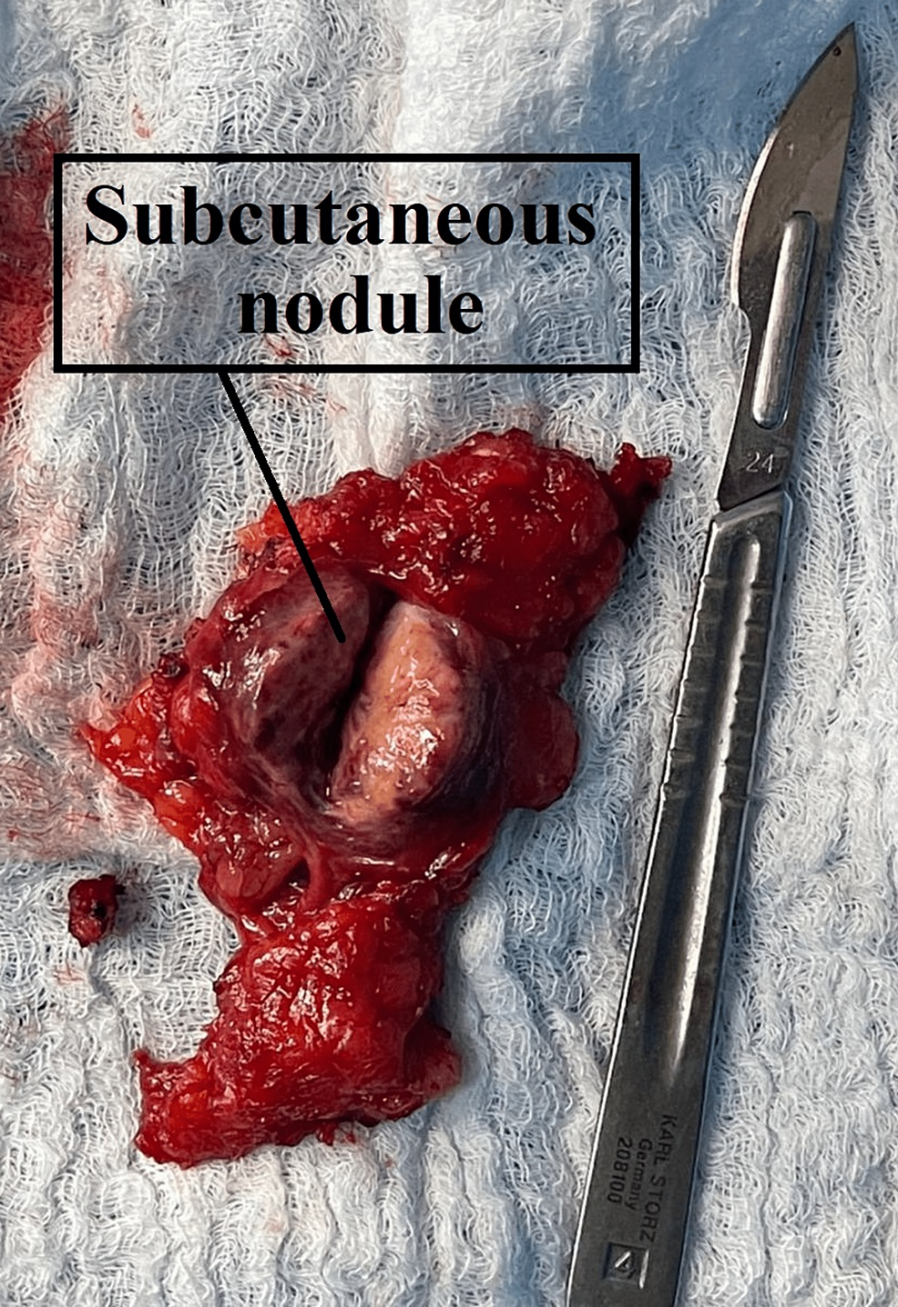
Subcutaneous nodule after dissection

During the operation, a vertical incision was created around the umbilicus and the wound was deepened down to the abdominal wall fascia by using electro-cautery. Then, the hernia sac was divided at the level of the fascia leaving it connected to the subcutaneous nodule. The umbilicus was later reversed and a purple endometrial tissue was observed implanted at its base (Figures 2, 3). After that, an incision was created to encompass the involved skin and the specimen (including skin, hernia sac, endometrioma) was pulled over and sent to the histopathology lab for assessment.

The hernia was repaired by using nylon 1, double layers. The first layer was continuous while the second was interrupted. Then, the subcutaneous tissue was sutured using 2-0 Vicryl and the umbilical skin was reconstructed using nylon 0 subcuticular. The patient underwent the surgical operation successfully. Later, she was referred to the gynecology department for further evaluation of any other endometriosis sites, through pelvic examination and ultrasound imaging; no abnormality was reported in this regard. Moreover, the histopathological assessment of the collected specimen confirmed the diagnosis of endometriosis. Finally, the patient was discharged well from the hospital. As a follow-up plan, the patient was informed about the risk of recurrence for both endometriosis and hernia. She was advised to maintain a high-fiber diet and to avoid heavy weight lifting and any other strenuous activity for at least four to six weeks after the surgery. Also, the patient was advised to follow up with a gynecologist within two to six weeks post-surgery and commence hormonal therapy.

## Discussion

Primary umbilical endometriosis is a rare medical condition and when associated with an underlying hernia, its diagnosis becomes challenging. Indeed, the diagnosis of umbilical endometriosis can be mistaken for a cyst, abscess, melanoma, lipoma, suture granuloma or a deposit of systemic malignancy [8]. Endometriosis should be suspected in all premenopausal females presenting with umbilical swelling, discharge, pain and cyclical bleeding from the umbilicus [5]. Many of these patients may have concomitant pelvic endometriosis. Therefore, it is recommended to use preoperative imaging before proceeding to an elective repair [2]. Magnetic resonance imaging (MRI) is still considered the modality of choice for the preoperative assessment of endometriosis. MRI has the capacity to outline the location and size of extra-pelvic endometriosis and also to eliminate any intra-abdominal expansion of the disease [9].

The histopathological appearance of an endometriosis section is usually characterized by the irregular glandular lumina that is embedded in the stroma with an elevated vascular and cellular component similar to the stroma of the functional endometrium [3]. Also, in a case studied, the examination of cutaneous endometriosis using epiluminescence microscopy revealed a special dermatoscopic feature of small red globular structures known as “red atolls” [10].

Surgical resection and hernia repair are the preferred treatment options for umbilical endometriosis associated with an underlying hernia. Additionally, hormonal therapy like gonadotropin-releasing hormone (GnRH) analogues or danazol can be used in severe cases [2,3,11]. Laparoscopic and open approaches can be used for the wide excision of an umbilical lesion and repair of hernia. During surgical resection, spillage should be avoided to prevent disease recurrence. Also, superficial therapies, such as thermocoagulation, are not recommended as it can predispose the patient to disease relapse [12].

In this case report, both clinical presentation and ultrasound imaging suggested that the patient had an umbilical nodule and an underlying hernia. The hernia defect was repaired primarily as its diameter was less than 2 cm. Mesh repair is recommended for recurrent or bigger hernias [13]. Several approaches are available for umbilical reconstruction depending on the size of the defect. Smaller defects, as in this case, can be closed by using interrupted absorbable sutures. On the other hand, larger defects can be sealed by flaps [2].

## Conclusions

Endometriosis is a common gynecological condition, but spontaneous umbilical endometriosis is very rare especially when associated with an underlying hernia. Moreover, the diagnosis of primary umbilical endometriosis associated with an underlying hernia is considered difficult. Therefore, the use of preoperative imaging is recommended for better preoperative planning. Surgical excision of the umbilical lesion with a hernia repair is considered the preferred treatment option.
